# High-frequency met-ocean observation dataset derived from the low-cost Marine Automatic Weather Station (MAWAS) platform at the Cirebon Coastal Area, Indonesia

**DOI:** 10.1016/j.dib.2026.113033

**Published:** 2026-06-28

**Authors:** Iwan Pramesti Anwar, Sonny Prayogo, Ayi Tarya, Faruq Khadami, Farrah Hanifah, Nining Sari Ningsih, Yazid Ridla, Ivonne M. Radjawane, Kim Namhoon, Mutiara R. Putri, Fikry Purwa Lugina

**Affiliations:** a Research Group of Applied Oceanography, Faculty of Earth Sciences and Technology, Institut Teknologi Bandung, Bandung 40132, Indonesia; b PT Samudra Sains Teknologi, Bandung, 40151, Indonesia; c Research Group of Sustainable Ocean Management, Faculty of Earth Sciences and Technology, Institut Teknologi Bandung, Bandung, 40132, Indonesia; d Korea-Indonesia Marine Technology Cooperation Research Center, Cirebon, 45611, Indonesia; e Korea Institute of Ocean Science and Technology, Busan, 49111, South Korea; f Laboratory of Applied Meteorology, Faculty of Earth Sciences and Technology, Institut Teknologi Bandung, Bandung, 40132, Indonesia

**Keywords:** Nearshore environmental monitoring, IoT sensor system, Real-time telemetry, Tidal water level, Tropical coastal atmosphere

## Abstract

This article describes a high-frequency coastal environmental dataset acquired by the MAWAS (Marine Automatic Weather Station) installed at Cirebon Jetty, Indonesia (6.716131°S, 108.572581°E). The dataset spans approximately seven months of continuous observation and includes seven environmental variables. They are timestamp, water level, air temperature, relative humidity, barometric pressure, wind speed, and wind direction. Observations were collected at an approximately 5 second sampling interval with near-real-time telemetry. The water-level series was geometrically corrected to convert raw ultrasonic sensor output into a calibrated water-level variable, and variable-level quality control was applied to remove outliers while preserving original timestamps. The dataset is publicly available through Mendeley Data and is suited for reuse in coastal meteorology, nearshore environmental monitoring, high-frequency time-series analysis, and real-time marine observation system evaluation in tropical coastal settings.

Specifications Table

**Table:** 

Subject	Earth & Environmental Sciences
Specific subject area	Coastal meteorology, nearshore environmental monitoring, high-frequency marine observation*.*
Type of data	Table Raw
Data collection	Observations were acquired using the ITB-MAWAS (Marine Automatic Weather Station) installed at Cirebon Jetty, Indonesia, during the official observation period from 1 October 2025 at 03:08:32 UTC to 4 May 2026 at 23:59:59 UTC. The platform used RS485 sensors for wind speed (WS200 A-3-N) https://cdsentec.com/wp-content/uploads/2022/06/WS200.pdf, wind direction (WD300 A-3) https://cdsentec.com/wp-content/uploads/2022/06/WD300.pdf, air temperature-humidity-barometric pressure (S-THP-01A) https://files.seeedstudio.com/Bazaar/product_pdf/101991101.pdf, and water level (RS485 750 cm Ultrasonic Level Sensor) https://files.seeedstudio.com/Bazaar/product_pdf/101991041.pdf. The ultrasonic water-level sensor was mounted at an installation reference distance of 338 cm above the deployment datum; raw ultrasonic distance measurements were converted to water level using water_level = 338 cm − raw_water_level. Sensors were connected to an ESP32 data logger and observations were sampled at an approximately 5-second interval. Data were transmitted in near real time to a remote server through a cellular modem using the MQTT protocol and archived as daily JSON files. For dissemination, daily files were merged into a single tabular CSV dataset. Variable-level quality control was applied using spike screening based on a rolling median and median absolute deviation, supported by conservative range checks; outliers were replaced with blank entries while preserving original timestamps. The final quality-controlled dataset contains 3298,281 timestamped records*.*
Data source location	Institut Teknologi Bandung Jetty at Pelindo Port Area, Cirebon, West Java, Indonesia; coordinates: 6.716131°S, 108.572581°E*.*
Data accessibility	Repository name: Mendeley Data (https://data.mendeley.com) Data identification number: 10.17632/h5smxr3kbk.1 Direct URL to data: https://data.mendeley.com/datasets/h5smxr3kbk/1
Related research article	‘none’*.*

## 1 Value of the Data

• This dataset provides rare high-frequency met-ocean observations from a tropical coastal environment in Indonesia, where continuous in situ monitoring data remain limited, particularly for under-documented coastal regions such as northern Java.
• The data demonstrates the capability of the low-cost ITB Marine Automatic Weather Station (MAWAS) platform to support long-term coastal environmental monitoring with near-real-time transmission, making it relevant for affordable ocean-observing system development.
• The approximately 5 s sampling interval enables detailed analysis of short-term variability in water level, air temperature, relative humidity, barometric pressure, wind speed, and wind direction, including rapid atmospheric and nearshore changes that are often missed by lower-frequency datasets.
• The quality-controlled and corrected water-level dataset can support coastal meteorology, nearshore hydrodynamics, sensor-network evaluation, high-frequency data-processing methods, and improved evidence-based coastal spatial planning in the Cirebon coastal area.

## 2 Background

Continuous met-ocean observations are needed to document temporal changes in coastal environments. This is particularly relevant in tropical coastal waters, where wind, tides, local weather, and nearshore water-level fluctuations may vary over short time scales [1,4]. In Indonesia, many coastal areas still have limited in situ monitoring because conventional meteorological and oceanographic instruments are costly to install, operate, and maintain [2,3]. This limitation affects the availability of local baseline observations for coastal hazard assessment, environmental monitoring, model validation, and spatial planning [4,5]. The motivation for compiling this dataset was to document high-frequency coastal observations from the Cirebon coastal area using a low-cost automatic observing platform.

This data article presents a quality-controlled met-ocean observation dataset collected by the Marine Automatic Weather Station (MAWAS) platform at the Cirebon coastal area, Indonesia. The dataset includes timestamped records of water level, air temperature, relative humidity, barometric pressure, wind speed, and wind direction. Observations were collected at an approximately 5 s sampling interval and transmitted in near real time. The dataset provides documented field observations from a low-cost coastal monitoring system in a region where continuous local measurements remain limited.

## 3 Data Description

**Fig. 1:**
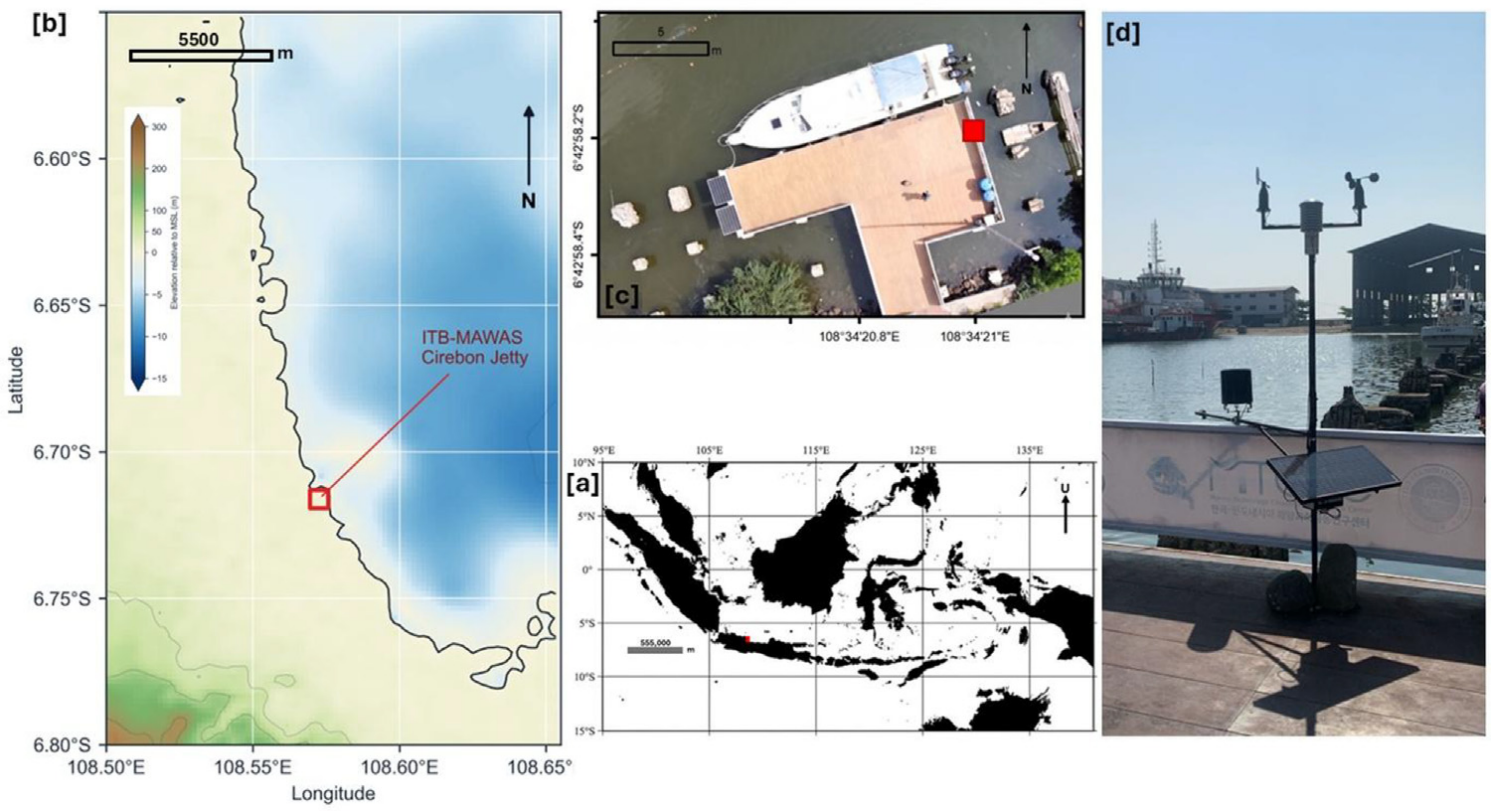
Location of the study site and MAWAS deployment. Indonesia map showing the Cirebon coastal area marked by a red box [a], the ITB Cirebon Jetty location marked by a red box [b], the Marine Automatic Weather Station deployment point at Cirebon Jetty marked by a filled red box [c], and photographs of the MAWAS setup at Cirebon Jetty [d].

The dataset described in this article consists of high-frequency met-ocean observations collected by the low-cost Marine Automatic Weather Station (MAWAS) platform deployed at the ITB Cirebon Jetty, Cirebon Coastal Area, Indonesia (Fig. 1). Cirebon coastal waters are located on the northern coast of Java and have been the focus of previous oceanographic, hydrographic, and tidal studies because of their importance for port activities, coastal resources, and environmental monitoring [[6], [7], [8]]. The present dataset complements these earlier studies by providing continuous fixed-station observations at a much higher temporal resolution [9].

The released dataset covers the official observation period from 1 October 2025 at 03:08:32 UTC to 4 May 2026 at 23:59:59 UTC. It contains 3298,281 timestamped records after quality control. The published variables are timestamp, water_level, temperature, humidity, pressure, wind_speed, and wind_direction. It located in [9] with file name ‘ITB-Marine Automatic Weather Station (MAWAS) at Cirebon Jetty.csv’. The instrument specifications and measurement ranges are summarized in Table 1. The raw measurements were transmitted in near real time from the field station to a remote server and archived as daily JSON files before being merged into tabular formats for dissemination.

**Table 1:** Marine automatic weather station parameters.

No.	Water Level (cm)	Air Temp. ( °C)	Humidity (%RH)	Barometric Pressure (hPa)	Wind Speed (m/s)	Wind Direction (°)
Range	28 to 750	−40 to 125	0 to 100 %	300 to 1100	0 to 30	0 to 360
Resolution	0.1	0.01	0.01 %	0.1	N/A	0.1
Accuracy	± (1 + S* × 0.3 %)	±0.1	±1.0 %	±0.15 relative; ±0.1 absolute	± (0.3 + 0.03 V*)	±1

⁎ S is the measured ultrasonic distance, and V is wind speed. For the released dataset, water level was transformed from the ultrasonic distance measurement using water_level = 338 cm - raw water_level. **Sensor models used in MAWAS: RS485 750 cm Ultrasonic Level Sensor, S-THP-01A, WS200 A-3-N, and WD300 A-3.

The water-level variable requires specific interpretation because the ultrasonic sensor originally measured the air-column distance between the sensor and the dynamic water surface. For the released dataset, this raw distance was transformed into water level using water_level = 338 cm − raw_water_level, where 338 cm represents the installation reference distance used for this deployment. This transformation allows the water-level series to be interpreted more directly as changes in water elevation at the jetty site.

**Fig. 2:**
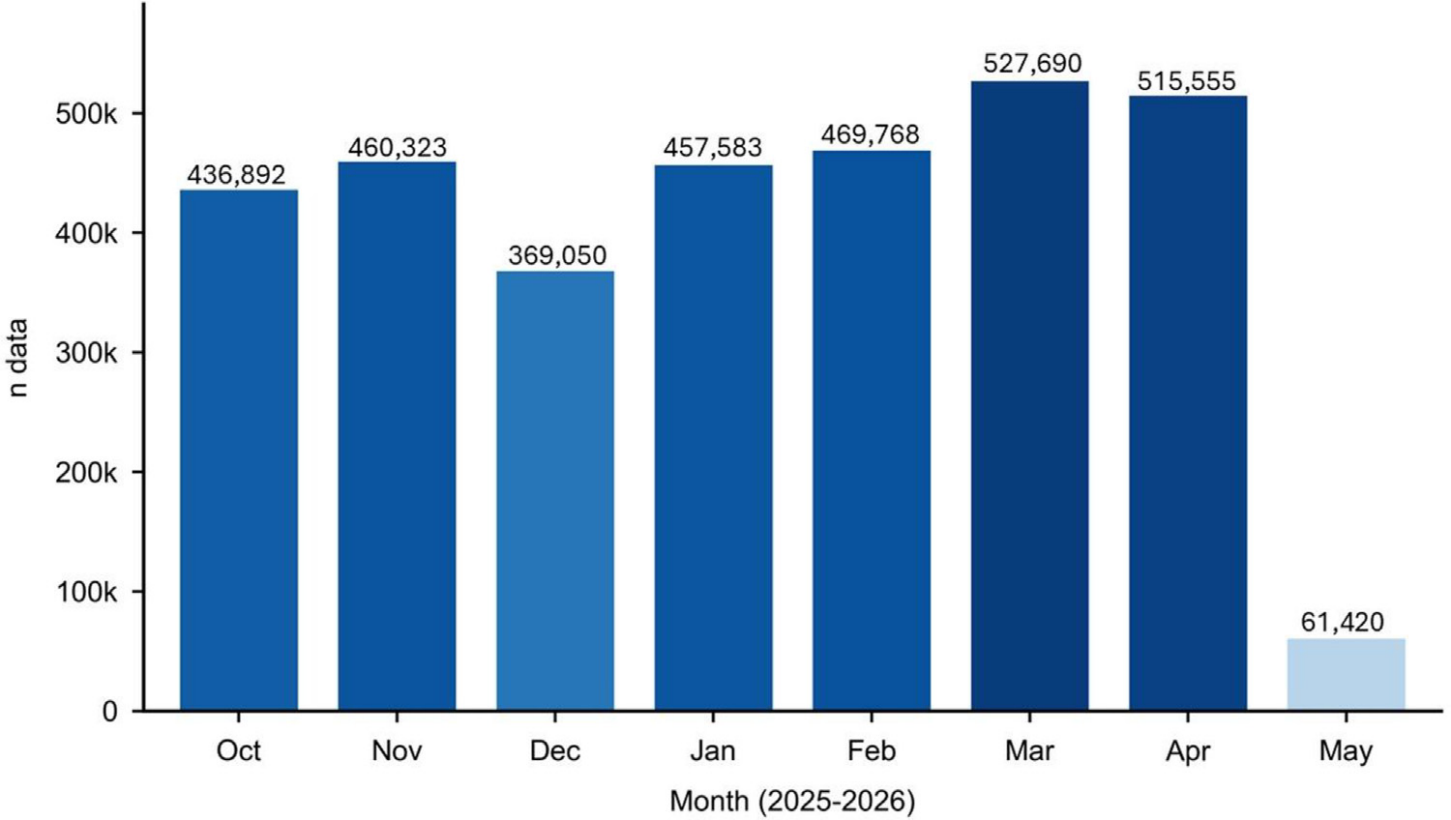
Monthly number of data recorded for all parameters.

The monthly number of valid records is shown in Fig. 2, which illustrates the temporal coverage of the dataset during the observation period. Most months contain dense records because the station sampled at approximately 5 s intervals. The lower count in May reflects the partial-month coverage ending on 4 May 2026. Summary statistics for each parameter are provided in Table 2. It includes the number of valid records, minimum and maximum values, the corresponding UTC time of occurrence, and the average value. After quality control, the water level ranged from 70.00 to 210.60 cm, air temperature from 22.85 to 35.21 °C, relative humidity from 40.98 to 100.00 %, barometric pressure from 1004.50 to 1015.80 hPa, and wind speed from 0.00 to 9.40 m/s.

**Table 2:** Data statistics for each parameter.

Parameter	N	Min.	Time of min. (UTC)	Max.	Time of max. (UTC)	Ave.
Water level (cm)	3295,719	70.00	2026–01–03 09:16:52	210.60	2025–12–04 00:46:46	137.55
Air temperature ( °C)	3298,279	22.85	2025–12–17 10:24:10	35.21	2025–10–26 04:44:15	27.70
Relative humidity (%)	3298,279	40.98	2025–10–04 04:08:38	100.00	2025–11–11 12:55:32	83.44
Barometric pressure (hPa)	3298,281	1004.50	2025–10–19 08:27:25	1015.80	2025–10–07 14:22:46	1010.55
Wind speed (m/s)	3298,001	0.00	2025–10–01 04:34:22	9.40	2026–01–21 07:52:44	0.74

**Fig. 3:**
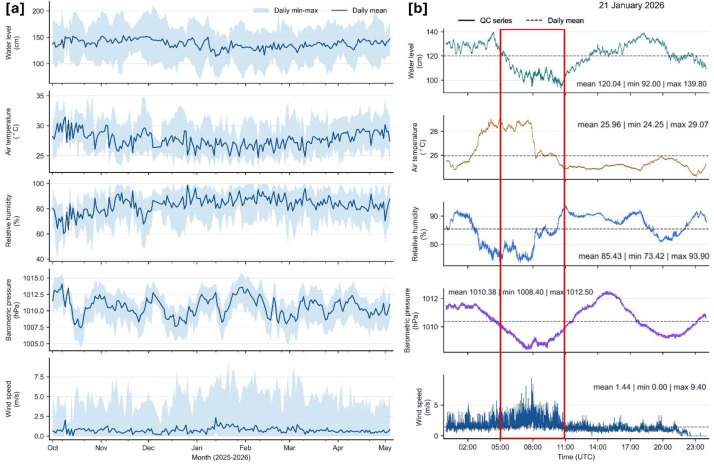
Daily mean time series, with minimum and maximum values shown as shaded areas, for all parameters except wind direction [a]. The period of maximum wind speed is highlighted by the red box and shown with the corresponding high-frequency observations of the other parameters [b].

To show the temporal behavior of the dataset, Fig. 3a presents daily average time series with minimum and maximum envelopes for all parameters except wind direction. This figure provides a compact overview of the day-to-day variability during the observation period. Fig. 3b highlights 21 January 2026, when the maximum wind speed was recorded, and shows the corresponding high-frequency responses of water level, air temperature, relative humidity, barometric pressure, and wind speed. This event-scale panel demonstrates the benefit of the approximately 5 s sampling interval for examining short-term variability that would be difficult to resolve from hourly or daily data.

**Fig. 4:**
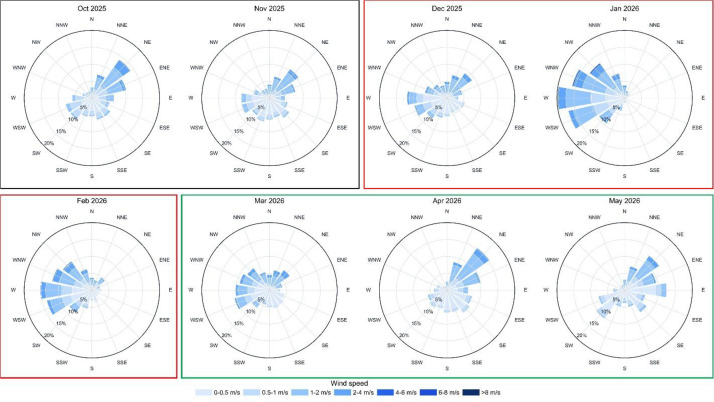
Monthly wind rose plots for the observation period. The black box indicates Transition Season II, the red boxes indicate the northwest monsoon period, and the green box indicates Transition Season I.

Wind-direction characteristics are summarized separately using monthly wind rose plots in Fig. 4, because wind direction is a circular variable and is not appropriately described by ordinary minimum, maximum, and arithmetic mean statistics. The wind rose panels show the monthly distribution of wind direction and wind-speed classes, with seasonal groupings indicated for Transition Season 2, the northwest monsoon, and Transition Season 1. Together, the time-series, statistical, and wind rose products provide a reusable dataset for coastal meteorology; nearshore water-level variability; low-cost sensor evaluation; quality-control method development; and evidence-based coastal spatial planning in the Cirebon Coastal Area.

## 4 Experimental Design, Materials and Methods

### 4.1 Experiment area

The observation was performed at ITB Cirebon Jetty, Cirebon Coastal Area, West Java, Indonesia, at 6.716131°S and 108.572581°E (Fig. 1). The station is located on the north coast of Java, facing the semi-enclosed waters of the Cirebon coast. This region is influenced by the local atmospheric forcing, tidal variability, port-related activities, and seasonal monsoon circulation. Several previous works around Cirebon waters have highlighted the importance of this area for hydrographic monitoring, tidal observation, port-channel evaluation, and coastal environmental management [[6], [7], [8]].

A MAWAS platform was installed on the jetty structure to continuously monitor the nearshore atmospheric and water-level conditions at a fixed point. The site was selected due to its direct access to the coastal water column and its suitability for instrument installation, maintenance, power supply management, and cellular data transmission. This location is also a strategic coastal monitoring point for northern Java, where continuous high frequency in situ datasets is scarce.

### 4.2 Method

The dataset was collected using the low-cost Marine Automatic Weather Station (MAWAS) platform. The MAWAS is considered low-cost in comparison with commercially available alternatives [[10], [11], [12], [13], [14]]. It has been developed for continuous met-ocean monitoring. The system integrates RS485-based sensors connected to an ESP32 data logger. MAWAS cover observation variables include water level, air temperature, relative humidity, barometric pressure, wind speed, and wind direction. The sensor types, measurement ranges, resolution, and accuracy are summarized in Table 1. Data was sampled at an approximately 5 s interval and transmitted in near real time to a remote server through a cellular modem using the MQTT protocol.

Each sensor was installed at a fixed position on the MAWAS platform structure on the jetty. The ultrasonic water-level sensor was mounted facing vertically downward at a reference height of 338 cm above the deployment datum, measuring the air-column distance to the dynamic water surface at an approximately 5-second interval. The wind speed and wind direction sensors were mounted at the top of the MAWAS mast to minimize flow obstruction. The air temperature, relative humidity, and barometric pressure sensor was housed in a radiation shield to reduce direct solar radiation exposure. All sensors sampled continuously and simultaneously at the same approximately 5-second interval without on-board averaging or aggregation. Each observation was individually timestamped in UTC at the moment of acquisition by the ESP32 data logger and transmitted immediately to the remote server via MQTT over a cellular modem. Prior to deployment, the wind direction sensor was calibrated to true north using a compass to ensure correct directional alignment. For all other parameters, sensor accuracy follows the manufacturer specifications as summarized in Table 1.

Raw observations were archived as daily JSON files on the server. For publication, the daily files were merged into a single time-series dataset covering the official observation period from 1 October 2025 at 03:08:32 UTC to 4 May 2026 at 23:59:59 UTC. The merged dataset was then converted into tabular formats for dissemination. The released variables are timestamp, water_level, temperature, humidity, pressure, wind_speed, and wind_direction.

The water-level measurement was processed before release because the ultrasonic sensor measured the air-column distance between the sensor and the dynamic water surface. This raw distance was converted into a water level using the following:

(1) water_level=338cm−rawwater_level

where 338 cm is the installation reference distance between the ultrasonic sensor and the deployment datum or seabed. This transformation makes the released water-level variable easier to interpret as relative water elevation at the jetty site.

Quality control was applied at a variable level. First, time stamps were standardized in UTC, and the dataset was restricted to the official observation period. Numeric variables were checked for valid ranges based on sensor specifications and physically reasonable limits. Then, the local spike detection was applied using a rolling median and median absolute deviation approach to identify short-duration outliers in the high-frequency records. After that, the values identified as outliers were replaced with blank entries, while the original timestamp sequence was preserved. This approach maintains the temporal structure of the dataset and allows users to distinguish missing or rejected values from valid observations.

After quality control, the final dataset contained 3298,281 records. Monthly data availability is summarized in Fig. 2, while descriptive statistics for each parameter are provided in Table 2. Daily mean, minimum, and maximum series were calculated to describe the temporal variability of the dataset (Fig. 3a). A high-wind event on 21 January 2026 was further extracted to show the behavior of the original high-frequency QC series during a short-term event (Fig. 3b). Wind direction and wind-speed distributions were summarized separately using monthly wind rose plots because wind direction is a circular variable and is better represented through directional frequency analysis than through arithmetic statistics (Fig. 4).

## Limitations

Several limitations of this dataset should be noted. First, the dataset represents observations from a single fixed station at Cirebon Jetty; spatial variability across the broader Cirebon coastal area cannot be inferred from this record alone, and multi-station deployments would be needed for spatially distributed analysis. Second, the water-level variable is referenced to an installation-specific datum (338 cm from the ultrasonic sensor to the deployment surface) rather than a standard geodetic or tidal datum, which limits direct comparison with official tide gauge records without an additional datum offset correction. Third, the observation period of approximately seven months (October 2025 to May 2026) covers one monsoon transition cycle, which may not be sufficient to characterise multi-year variability or long-term trends. Fourth, real-time data transmission depends on cellular network availability, and any network interruption results in data gaps, as reflected in the lower record count for December 2025. These limitations also represent opportunities for future work, including multi-station deployments, datum-referenced water-level integration, independent sensor validation, and extended long-term observation.

## Ethics Statement

The authors declare that the present work did not include experiments on human subjects and/or animals, or any data collected from social media platforms.

## CRediT Author Statement

**Iwan Pramesti Anwar:** Conceptualization, Project administration, Writing – original draft. **Sonny Prayogo:** Investigation, Software, Data curation. **Ayi Tarya:** Supervision, Writing - Review & Editing. **Faruq Khadami:** Data curation, Writing – review & editing. **Farrah Hanifah:** Visualization, Writing – review & editing. **Nining Sari Ningsih:** Supervision, Writing – review & editing. **Yazid Ridla:** Investigation, Resources. **Ivonne M. Radjawane:** Funding acquisition, Supervision. **Kim Namhoon:** Writing – review & editing. **Mutiara R. Putri:** Supervision, Formal analysis. **Fikry Purwa Lugina:** Data curation, Validation.

## Declaration of generative AI and AI-assisted technologies in the manuscript preparation process

During the preparation of this work the author(s) used OpenAI’s ChatGPT/Codex and Quillbot in order to assist with language editing, grammar refinement, manuscript structuring, figure-caption polishing, and code support for data processing and visualization. After using this tool/service, the author(s) reviewed and edited the content as needed and take(s) full responsibility for the content of the published article.

## Data Availability

Mendeley DataRaw data from high-frequency observations of the ITB-MAWAS (Marine Automatic Weather Station) at Cirebon Jetty (Original data).

## References

[1] (2024). https://wmo.int/guide-instruments-and-methods-of-observation-wmo-no-8-0.

[2] Bernardes G.F.L.R., Ishibashi R., Ivo A.A.S., Rosset V., Kimura B.Y.L. (2023). Prototyping low-cost automatic weather stations for natural disaster monitoring. Digit. Commun. Netw..

[3] Albaladejo C., Soto F., Torres R., Sánchez P., López J.A. (2012). A low-cost sensor buoy system for monitoring shallow marine environments. Sensors.

[4] Pineau-Guillou L., Lazure P. (2025). A high-frequency dataset of sea level observations from low-cost pressure sensors. Sci. Data.

[5] Ehler C., Douvere F. (2009). https://repository.oceanbestpractices.org/handle/11329/459.

[6] Badriana M.R., Nur A.A., Hidayatullah A.I., Prastyo A.W., Bernawis L.I., Jeon C., Radjawane I.M., Park H. (2023). Seasonal monitoring of ocean parameter over green mussel cultivation area in west part of Cirebon seawater. Indones. J. Mar. Sci..

[7] Badriana M.R., Nur A.A., Hidayatullah A.I., Abdurrahman U., Jeon C.K. (2024). Ocean parameter and hydrographic measurement around Cirebon port channel. IOP Conf. Ser..

[8] Badriana M.R., Abdurrahman U., Nur A.A., Jeon C., Radjawane I.M., Park H. (2024). Tidal characteristics analysis utilizing radar tide gauge in Cirebon seawater. J. Ilmu dan Teknol. Kelaut. Trop..

[9] Anwar Iwan Pramesti, Prayogo Sonny (2026). Raw data from high-frequency observations of the ITB-MAWAS (Marine Automatic Weather Station) at Cirebon Jetty. Mendeley Data.

[10] Nubila, “Nubila Marco weather station,” [Online]. Available: https://nubila.ai/order. [Accessed: June 22nd, 2026].

[11] RainWise, (2026). “MK-III Internet Weather Station Package,” [Online]. Available: https://rainwise.com/the-mk-iii-internet-package. [Accessed: June 22nd, 2026].

[12] Kestrel Instruments, (2026). “KestrelMet 6000 Agricultural Weather Station,” [Online]. Available: https://kestrelmet.com/weather-stations. [Accessed: June 22nd, 2026].

[13] Davis Instruments, (2026). “Davis Vantage Pro2 Weather Station,” [Online]. Available: https://www.davisinstruments.com/products/weatherlink-console. [Accessed: June22nd, 2026].

[14] PT Majuma Panmandiri, (2026). “Valeport VRS-20 radar level sensor,” [Online]. Available: https://www.majumapanmandiri.co.id/index.php?route=product/product&product_id=1088. [Accessed: June 22nd, 2026].

